# Increased Blood Concentrations of Malondialdehyde in *Plasmodium* Infection: A Systematic Review and Meta-Analysis

**DOI:** 10.3390/antiox12081502

**Published:** 2023-07-27

**Authors:** Onchuma Mueangson, Aongart Mahittikorn, Nsoh Godwin Anabire, Wanida Mala, Manas Kotepui

**Affiliations:** 1 School of Allied Health Sciences, Walailak University, Tha Sala, Nakhon Si Thammarat 80160, Thailand; 2 Department of Protozoology, Faculty of Tropical Medicine, Mahidol University, Bangkok 10400, Thailand; 3 Department of Biochemistry and Molecular Medicine, School of Medicine, University for Development Studies, Tamale P.O. Box TL1350, Ghana; 4 West African Centre for Cell Biology of Infectious Pathogens (WACCBIP), Department of Biochemistry, Cell and Molecular Biology, University of Ghana, Accra P.O. Box LG 54, Ghana

**Keywords:** malondialdehyde, MDA, lipid peroxidation, oxidant, antioxidant, malaria

## Abstract

Several studies have evaluated the relationship between malondialdehyde (MDA) concentrations and *Plasmodium* infections; however, the findings remain inconclusive. This study synthesized differences in MDA concentrations among patients with different levels of clinical severity, uninfected controls, and different *Plasmodium* species. The research protocol was registered in PROSPERO (CRD42023393540). Systematic literature searches for relevant studies were performed using the Embase, MEDLINE, Ovid, ProQuest, PubMed, Scopus, and Google Scholar databases. Qualitative and quantitative syntheses (meta-analyses) of distinct MDA concentrations between the disease groups were performed. Twenty-three studies met the eligibility criteria and were included in the systematic review. Overall, MDA concentrations were significantly elevated in participants with malaria relative to uninfected controls (*p* < 0.01, Cohen d: 2.51, 95% confidence interval (CI): 1.88–3.14, I^2^: 96.22%, 14 studies). Increased MDA concentrations in participants with malaria compared with uninfected controls were found in studies that enrolled patients with *P. falciparum* malaria (*p* < 0.01, Cohen d: 2.50, 95% CI: 1.90–3.10, I^2^: 89.7%, 7 studies) and *P. vivax* malaria (*p* < 0.01, Cohen d: 3.70, 95% CI: 2.48–4.92, I^2^: 90.11%, 3 studies). Our findings confirm that MDA concentrations increase during *Plasmodium* infection, indicating a rise in oxidative stress and lipid peroxidation. Thus, MDA levels can be a valuable biomarker for evaluating these processes in individuals with malaria. However, further research is necessary to fully elucidate the intricate relationship between malaria, antioxidants, oxidative stress, and the specific role of MDA in the progression of malaria.

## 1. Introduction

Lipid peroxidation is involved in the pathogenesis of various tissue injuries [1,2]. Oxidative stress facilitates lipid peroxidation in the cell membrane, resulting in the formation of harmful substances such as aldehydes, including malondialdehyde (MDA) and 4-hydroxy-2-nonenal, as well as other toxic compounds [3]. MDA, a well-known secondary product of lipid peroxidation, can serve as a biomarker for cell membrane damage [4]. As a colorless liquid and potent oxidizing agent, MDA is naturally produced in response to oxidative stress [5]. Thus, elevated MDA levels indicate increased oxidative stress through the process of lipid peroxidation [6]. Increased levels of lipid peroxidation products have been linked to several human illnesses, including malaria [7,8,9].

In humans, malaria is caused by the infection of one or more of the five *Plasmodium* parasites (predominantly *Plasmodium falciparum* and *P. vivax*) through bites from *Anopheles* spp. mosquitoes [10]. Malaria remains one of the leading causes of death among children under the age of 5 years in Africa [11]. In response to *Plasmodium* infection, host cells release reactive oxygen species (ROS), which not only clear the parasite but also damage host cells and tissues, leading to severe pathologies [12]. Many studies have reported elevated levels of oxidative stress markers, including MDA, in malaria patients [13,14,15,16,17]. Two basic processes lead to oxidative stress in malaria. First, the parasite structurally damages erythrocytes during its replication cycle, altering characteristics such as stiffness, viscosity, and volume [18,19] and resulting in oxidative stress due to the destruction of host hemoglobin. Second, the host immune system launches a number of defense mechanisms in response to oxidative stress, culminating in the release of free radicals by activated macrophages to combat the parasite [20,21]. The production of reactive hydroxyl radicals due to mitochondrial oxidative stress has been linked to liver apoptosis in animals with malaria [22,23]. Human erythrocytes infected with malaria parasites show increased levels of oxidative stress [24,25], which impacts disease severity by causing red cell lysis, leading to anemia and a reduction in iron concentration.

The relationship between MDA levels and *Plasmodium* infections has been investigated in the literature, but the results are inconsistent. Furthermore, MDA concentrations may vary according to the clinical severity of the disease or *Plasmodium* species. Therefore, this systematic review synthesized differences in MDA concentrations between participants with malaria and uninfected controls. Additionally, we examined variations in MDA levels between patients with severe and nonsevere malaria, as well as between patients with *P. falciparum* and *P. vivax* malaria.

## 2. Methods

### 2.1. Search Strategy

This research protocol was registered in PROSPERO (CRD42023393540). The study was conducted and reported according to the PRISMA protocol for reporting systematic reviews and meta-analyses [26]. Systematic searches of the literature for relevant studies published up to 18 January 2023 were performed using the Embase, MEDLINE, Ovid, ProQuest, PubMed, and Scopus databases. The key terms used in the search strategy were (Propanedial OR Malonyldialdehyde OR Malonaldehyde OR Malonylaldehyde OR “Sodium Malondialdehyde”) AND (malaria OR Plasmodium OR “Remittent Fever” OR “Marsh Fever” OR Paludism). For the search in PubMed, the MeSH terms were identified in the search strategy as “(((malaria) OR (malaria [MeSH Terms])) OR (Plasmodium)) OR (Plasmodium [MeSH Terms]) AND (Malonyldialdehyde) OR (Malonyldialdehyde[MeSH Terms]).” The search strategy used in different databases differed slightly according to the format of each database (Table S1). The publication date and language were not restricted. The literature searches were also conducted using Google Scholar to ensure that no relevant articles were overlooked and to identify additional relevant articles.

### 2.2. Inclusion and Exclusion Criteria

The eligibility criteria for the review were determined using the patient, intervention, comparison, outcome (PICO) framework [27] as follows: P, participants with malaria; I, none; C, uninfected controls; and O, MDA concentration. The following articles were considered eligible for this review: (i) studies in which malaria cases were diagnosed using a single method (or combination of methods), including microscopy, a rapid diagnostic test, serology, or molecular methods; (ii) studies that evaluated MDA concentration in malaria cases using thiobarbituric acid assay [28]; and (iii) studies that recruited healthy or febrile participants as uninfected controls. Reviews, systematic reviews, meta-analyses, animal studies, in vitro studies, comments, letters to the editor, and case reports were excluded.

### 2.3. Study Selection and Data Extraction

The articles were input into EndNote version 20.0 (Clarivate Analytics, Philadelphia, PA, USA), duplicates were removed, and the remaining articles were checked for eligibility. After removing irrelevant articles, the full texts of the remaining articles were examined to see if they met the requirements. Studies that failed to meet the eligibility requirements were then removed, and a clear explanation was given. Data on the study and participant characteristics and the PICO criteria were extracted from each study, including the name of the first author, publication year, study design and area, the number of participants and age range, MDA concentrations in malaria patients and uninfected controls, *Plasmodium* identification methods, and MDA concentration methods. Study selection and data extraction were performed independently by two authors (O.M. and M.K.), and any disagreements were discussed with another author (A.M.).

### 2.4. Quality Assessment

The Strengthening the Reporting of Observational Studies in Epidemiology (STROBE) checklists for observational studies were used to assess the quality of the studies [29]. These checklists are used to score a maximum of 22 points for each study across all sections of the article: title and abstract, 1; introduction, 2; method, 9; results, 5; discussion, 4; and other information, 1. The quality assessment was performed independently by two authors (O.M. and W.M.), and any disagreements were discussed to reach a final agreement. Studies with a STROBE score percentage of <50%, 50–75%, and >75% were considered low-, moderate-, and high-quality studies, respectively [30] (Table S2).

### 2.5. Data Syntheses

Qualitative synthesis was used to explain qualitative differences in MDA concentrations in the disease groups (malaria vs. uninfected controls, severe vs. nonsevere malaria, and *P. falciparum* vs. *P. vivax* infection). Quantitative synthesis (meta-analysis) of distinct MDA concentrations between the disease groups (malaria vs. uninfected controls, severe vs. nonsevere malaria, and *P. falciparum* vs. *P. vivax* infection) was performed using the random-effect model [31]. I^2^ statistics were utilized to assess between-study heterogeneity, with I^2^ values <50, 50–75, and >75 categorized as having low, moderate, and high between-study heterogeneity, respectively [30]. Meta-regression and subgroup analyses were performed to investigate the potential source(s) of between-study heterogeneity. The funnel plot, Egger test, and trim-and-fill methods were used to determine the publication bias [32]. The leave-one-out meta-analysis method was used to determine whether one study affected the overall effect estimate [33]. Statistical analyses were performed using STATA 17.0 (StataCorp, College Station, TX, USA).

## 3. Results

### 3.1. Search Results

**Figure 1:**
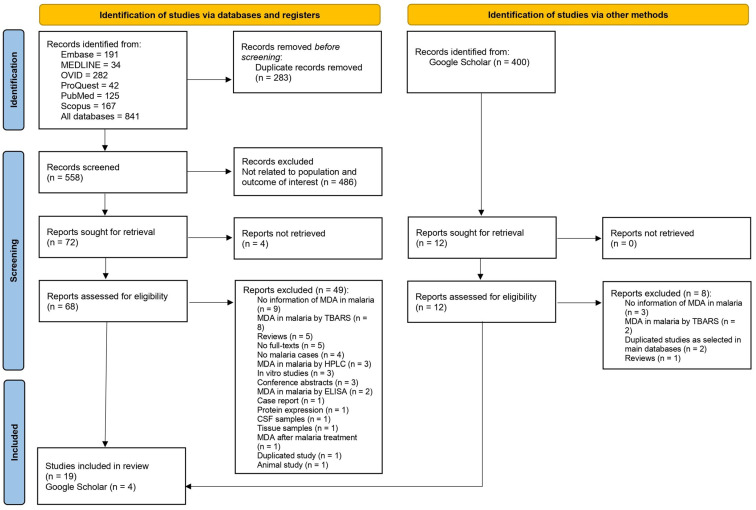
Study selection diagram.

A total of 841 articles were extracted from six databases, including Embase (n = 191), MEDLINE (n = 34), Ovid (n = 282), ProQuest (n = 42), PubMed (n = 125), and Scopus (n = 167). After removing 283 duplicate articles, the remaining 558 were reviewed, and a further 486 irrelevant articles were removed. The remaining 72 full-text articles were examined against the eligibility criteria, and those that failed to meet them were excluded with a clear explanation, leaving 19 articles [7,34,35,36,37,38,39,40,41,42,43,44,45,46,47,48,49,50,51] for inclusion in our review. Additionally, four articles [52,53,54,55] were identified from Google Scholar that also met the inclusion criteria. Thus, a total of 23 studies [7,34,35,36,37,38,39,40,41,42,43,44,45,46,47,48,49,50,51,52,53,54,55] were included for review (Figure 1).

### 3.2. Summary Characteristics of the Included Studies

Of the 23 included studies, most (65.2%) were published between 2010 and 2022 and had a cross-sectional design (91.2%). The majority were performed in African countries (41.8%) and Asian countries (41.8%), with Nigeria and India the most representative countries in Africa (63.6%) and Asia (72.7%), respectively. Most studies enrolled patients infected with *P. falciparum* (47.8%), and most study participants were adults (39.1%). Over half of the studies used microscopy alone for *Plasmodium* detection (52.2%) (Table 1).

### 3.3. Quality of the Included Studies

Based on the STROBE checklist, seven studies (21.7%) were of high quality, and the remainder (78.3%) were of moderate quality. None of the studies were of low quality (Table S3). To investigate whether the quality of the studies affected the pooled effect estimate, a meta-regression analysis was performed using the study’s quality as a covariate.

### 3.4. MDA Concentrations in Malaria and Uninfected Controls

A qualitative synthesis showed increased MDA concentrations in participants with malaria compared with uninfected controls in 19 studies [7,34,36,37,38,39,40,41,42,43,44,45,47,48,49,50,53,54,55]. Two studies [35,46] showed no difference in MDA concentrations between malaria patients and uninfected controls. Two studies did not report a difference in MDA levels of malaria patients compared with uninfected controls [51,52]. Five studies reported a significant positive correlation between MDA concentration and parasite density [7,45,48,49,52] (Table 2).

**Figure 2:**
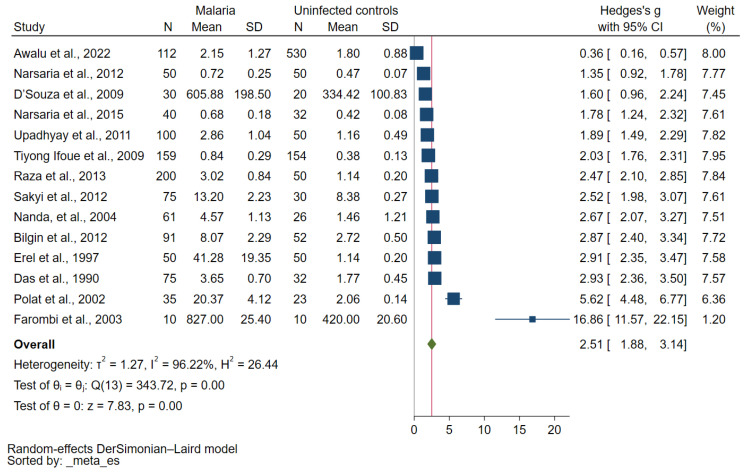
Forest plot showing differences in MDA concentration between participants with malaria and uninfected controls. Blue square, MDA concentration; green diamond, pooled Cohen d; gray line, no difference in MDA concentration between the two groups; red line, pooled Cohen d. Test of overall effect (*p* = 0.00) indicates increased MDA concentration in malaria patients compared with uninfected controls [34,36,37,38,40,42,43,44,47,48,49,50,53,54]. Abbreviations: N, number of participants; mean, mean MDA concentrations (using Cohen d as an effect estimate, any unit of mean MDA concentration can be used in the meta-analysis); SD, standard deviation; CI, confidence interval.

Fourteen studies reported quantitative data; thus, they were used for quantitative synthesis [34,36,37,38,40,42,43,44,47,48,49,50,53,54]. Overall, MDA concentrations were increased in participants with malaria compared with uninfected controls (*p* < 0.01, Cohen d: 2.51, 95% confidence interval (CI): 1.88–3.14, I^2^: 96.22%, 14 studies; Figure 2).

**Figure 3:**
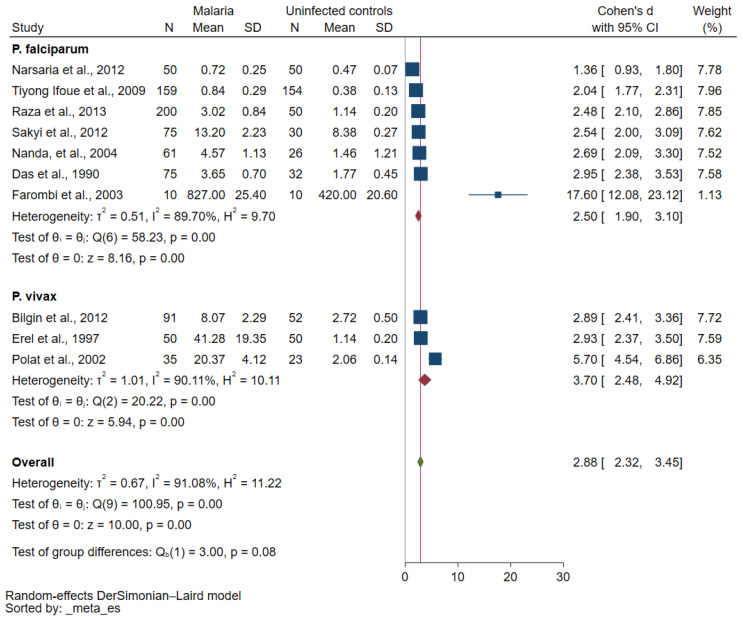
Forest plot of differences in MDA concentration between participants with malaria and uninfected controls (stratified by *Plasmodium* species) [36,38,40,42,43,47,48,49,53,54]. Blue square, MDA concentration; green diamond, pooled Cohen d; crimson diamond, pooled Cohen d in each subgroup; gray line, no difference in MDA concentration between the two groups; red line, pooled Cohen d. Abbreviations: N, number of participants; mean, mean MDA concentrations (using the Cohen d as an effect estimate, any unit of mean MDA concentrations can be used in the meta-analysis); SD, standard deviation.

**Figure 4:**
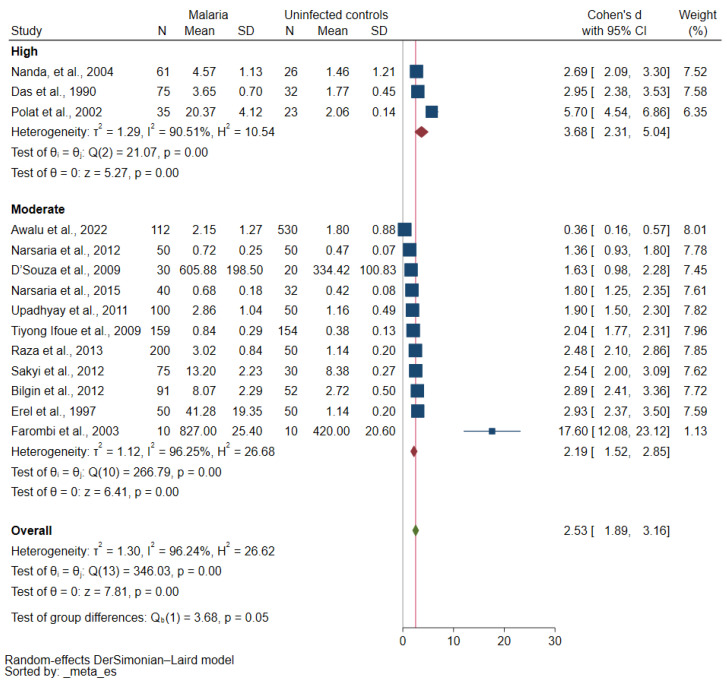
Forest plot of differences in MDA concentration between participants with malaria and uninfected controls (stratified by the study’s quality) [34,36,37,38,40,42,43,44,47,48,49,50,53,54]. Blue square, MDA concentration; green diamond, pooled Cohen d; crimson diamond, pooled Cohen d of subgroups; gray line, no difference in MDA concentration between the two groups; red line, pooled Cohen d. Abbreviations: N, number of participants; mean, mean MDA concentrations (using the Cohen d as an effect estimate, any unit of mean MDA concentrations can be used in the meta-analysis); SD, standard deviation; CI, confidence interval.

Meta-regression analyses of publication year, participants, study design, continent, *Plasmodium* species, assays for *Plasmodium* identification, and study quality showed that the *Plasmodium* species and the study’s quality may affect the pooled effect estimate (*p* value was borderline significant, Table S4). Further subgroup analyses using *Plasmodium* species and the study’s quality were performed. The subgroup analysis of *Plasmodium* species showed no subgroup difference (*p* = 0.08, Figure 3). Increased MDA concentrations in participants with malaria compared with uninfected controls were reported in studies that enrolled patients with *P. falciparum* malaria (*p* < 0.01, Cohen d: 2.50, 95% CI: 1.90–3.10, I^2^: 89.7%, seven studies) and those with *P. vivax* malaria (*p* < 0.01, Cohen d: 3.70, 95% CI: 2.48–4.92, I^2^: 90.11%, three studies). The subgroup analysis of the study’s quality revealed a borderline subgroup difference (*p* = 0.05, Figure 4). Increased MDA concentrations in malaria cases compared with uninfected controls were found in studies of high quality (*p* < 0.01, Cohen d: 3.68, 95% CI: 2.31–5.04, I^2^: 90.51%, 3 studies) and moderate quality (*p* < 0.01, Cohen d: 2.19, 95% CI: 1.52–2.85, I^2^: 96.25%, 11 studies).

### 3.5. MDA Concentrations in Severe and Nonsevere Malaria

A qualitative synthesis showed increased MDA concentrations in severe and nonsevere malaria patients in a study by Sakyi et al. [48]. Regarding patients with severe malaria with different complications, Villaverde et al. showed no difference in MDA concentrations between children with malaria retinopathy-positive cerebral malaria and children with malaria retinopathy-negative cerebral malaria [51]. The analysis of differences in MDA concentrations between patients with severe and nonsevere malaria using the meta-analysis approach could not be performed due to an insufficient number of studies.

### 3.6. MDA in P. falciparum and P. vivax Malaria

In two studies [37,50], a qualitative synthesis showed increased MDA concentrations in *P. falciparum* malaria compared with *P. vivax* malaria. Lower MDA concentrations in *P. falciparum* malaria compared with *P. vivax* malaria have been reported [41]. Differences in MDA concentrations between patients with *P. falciparum* and *P. vivax* malaria using the meta-analysis approach could not be determined due to the insufficient number of studies.

### 3.7. Sensitivity Analysis and Publication Bias

**Figure 5:**
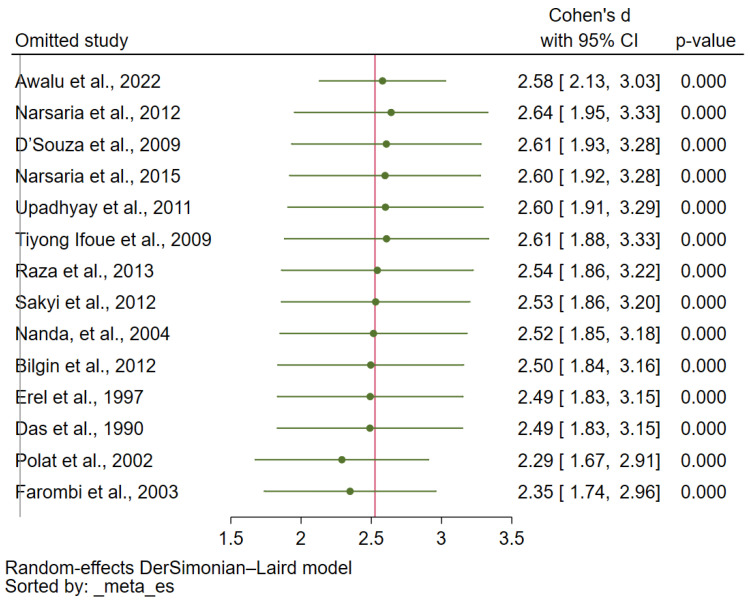
Leave-one-out meta-analysis demonstrating the meta-analysis results (malaria vs. uninfected controls) [34,36,37,38,40,42,43,44,47,48,49,50,53,54] after one study was removed at a time and the meta-analysis was rerun. Green dot, pooled Cohen d; gray horizontal line, 95% CI of Cohen d, red line, pooled Cohen d. Abbreviations: CI, confidence interval.

**Figure 6:**
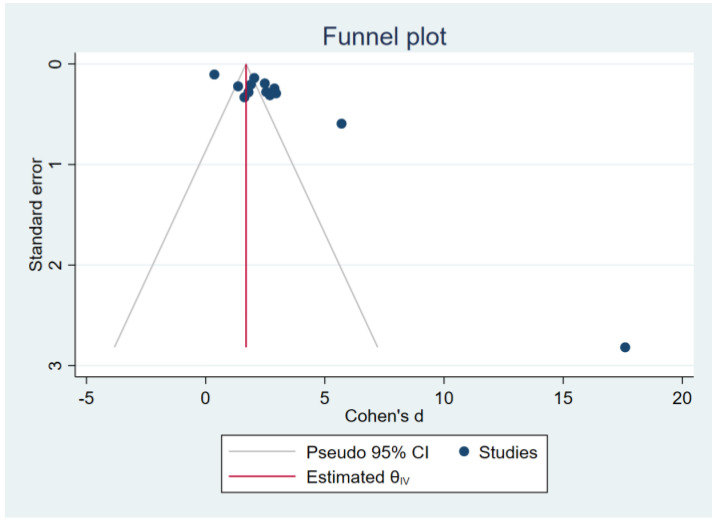
Funnel plot presenting the Cohen d of individual studies (blue dot) on the left and right sides of the pooled Cohen d (red line).

In all reruns using the leave-one-out meta-analysis method, participants with malaria had higher MDA concentrations than the uninfected controls (Figure 5). The funnel plot in the comparison analysis of MDA concentrations between participants with malaria and uninfected controls showed asymmetry (Figure 6). The Egger test revealed a statistically significant small-study effect (*p* < 0.01). The trim-and-fill analysis showed increased MDA concentrations in malaria cases compared with uninfected controls (Cohen d: 1.70, 95% CI: 1.58–1.81).

## 4. Discussion

MDA is a well-known marker of oxidative stress in several diseases [7,8,9]. It is generated by free radicals that cause membrane lipid peroxidation and has been shown to deplete antioxidant levels, increase proinflammatory cytokines, and increase oxidative stress [56]. The present study confirmed previous reports of increased MDA levels in participants with malaria compared with uninfected controls [7,34,35,36,37,38,39,40,41,42,43,44,45,47,48,49,50,53,54,55]. It is possible that lipid peroxidation on erythrocyte membranes—which are vulnerable to oxidative damage—is the cause of the elevated MDA levels found in malaria patients. The lipid peroxides released into the bloodstream by erythrocyte membranes that have suffered oxidative damage are degraded, resulting in increased MDA concentrations [57]. These findings suggest that *Plasmodium* infection in humans results in the host’s release of ROS, which aid in parasite clearance [12]. However, high levels of these ROS can harm host cells and tissues, predisposing them to severe disease outcomes [12]. The primary cause may be the malaria parasite’s dependence on hemoglobin as a source of vital amino acids necessary for growth and maintenance during the erythrocytic stage of its life cycle [58]. As a result, the extent of hemoglobin degradation depends on malaria severity. A low hemoglobin level suggests increased oxidative stress, which is reflected by an increased level of MDA but a decreased level of antioxidants [59]. Thus, MDA levels can be used to measure the disease severity in malaria patients along with other evaluations.

The subgroup meta-analysis revealed elevated MDA concentrations in cases of malaria caused by *P. falciparum* or *P. vivax*. Due to the limited number of studies investigating MDA levels in both of these *Plasmodium* species, the results exhibit significant heterogeneity and lack clarity. Specifically, only two studies [37,50] reported higher MDA concentrations in *P. falciparum* than in *P. vivax* infections. This disparity may be attributed to the more severe nature of *P. falciparum* infection, which induces greater oxidative stress in the host cells. However, another study found lower MDA concentrations in *P. falciparum* malaria than in *P. vivax* malaria [41]. This discrepancy may be explained by the increased susceptibility of patients with *P. vivax* malaria to oxidative stress, potentially due to lower levels of ascorbic acid [53]. Overall, the findings of this study are consistent with previous research indicating similar MDA concentrations in malaria caused by both *Plasmodium* species, regardless of parasitemia [60]. The subgroup meta-analysis demonstrated increased MDA concentrations in malaria cases, regardless of whether the quality of the included studies was high or moderate. Furthermore, when combined with the sensitivity analysis, the results strongly suggest that malaria leads to an excessive accumulation of MDA, which is indicative of oxidative stress. This conclusion is supported by robust findings in high-quality studies, affirming that *Plasmodium* infections induce oxidative stress-related MDA buildup.

The presence of MDA in malaria-infected individuals indicates the occurrence of lipid peroxidation and oxidative stress. Notably, MDA is one of many markers used to assess oxidative stress and lipid peroxidation, and its measurement alone may not provide a comprehensive understanding of the overall oxidative status in malaria. Furthermore, the association between MDA and the antioxidant system is an important aspect to consider in malaria. The antioxidant system is crucial to maintain the balance between ROS production and elimination [61]. It consists of enzymatic and nonenzymatic antioxidants that work together to neutralize and scavenge ROS, thereby protecting cells and tissues from oxidative damage [61,62]. Several studies investigating the relationship between MDA and the antioxidant system in the context of malaria showed that the activities of various antioxidant enzymes, such as superoxide dismutase (SOD), glutathione peroxidase, and catalase, were decreased during malaria [17,63,64]. A longitudinal birth cohort study reported that several polymorphisms in antioxidant enzymes, including glutathione reductase, glutamylcysteine synthetase, glutathione S-transferase P1, haem oxygenase 1, and SOD2, were associated with the oxidative stress status of children [65]. Since these enzymes are important for eliminating ROS and maintaining redox homeostasis, their decreased activity during malaria can impair the antioxidant defense system, leading to increased oxidative stress and lipid peroxidation, which contribute to MDA formation. Furthermore, the depletion of nonenzymatic antioxidants, including reduced glutathione, vitamin C, and vitamin E, during malaria has been reported [66,67]. These antioxidants directly scavenge ROS and help to regenerate enzymatic antioxidant activity. Reductions in their levels during malaria can impair the overall antioxidant capacity of the system, leading to increased lipid peroxidation and MDA formation.

Our systematic review and meta-analysis had some limitations. First, there was heterogeneity in MDA levels between the studies included in the meta-analysis. This heterogeneity might potentially be influenced by the very wide range of MDA levels due to differences in the quality of the included studies, as demonstrated by the subgroup analysis. Furthermore, MDA levels could be potentially influenced by characteristics of the diseased cohorts, such as the age of participants, the timing, and disease severity. Second, publication bias was observed in the meta-analysis of MDA concentrations between malaria patients and uninfected controls due to the small number of studies.

## 5. Conclusions

Our study confirms that MDA concentrations increase in cases of *Plasmodium* infection and are independent of the *Plasmodium* species (*P. falciparum* and *P. vivax*), at least for the limited studies included in this meta-analysis. The measurement of MDA levels can serve as a useful biomarker to evaluate oxidative stress and lipid peroxidation in individuals with malaria. These findings suggest that MDA concentrations can be suitably tracked as a potential indicator of *Plasmodium* infection. However, further research is necessary to fully understand the relationship between malaria, oxidative stress, and the role of MDA in the disease.

## Figures and Tables

**Table 1:** Summary characteristics of the included studies.

Characteristics	N (23 Studies)	%
Publication year		
2010–2022	15	65.2
2000–2009	6	26.1
Before 2000	2	8.70
Study design		
Cross-sectional	21	91.2
Case–control	1	4.35
Cohort	1	4.35
Study area		
Africa	**11**	**41.8**
Nigeria	7	63.6
Cameroon	2	18.2
Uganda	1	9.10
Ghana	1	9.10
Asia	**11**	**41.8**
India	8	72.7
Turkey	3	27.3
South America	**1**	**4.35**
Brazil	1	
*Plasmodium* species		
*P. falciparum*	11	47.8
*P. vivax*	4	17.4
* Plasmodium* spp.	8	34.8
Participants		
Adults	9	39.1
Children	6	26.1
Pregnant women	3	13.0
Patients with malaria (unspecified age)	3	13.0
Pregnant and nonpregnant women	2	8.70
Methods of *Plasmodium* detection		
Microscopy	12	52.2
Microscopy with other methods	3	13.0
PCR	1	4.35
RDT	1	4.35
Unspecified	6	26.1

Abbreviations: PCR, polymerase chain reaction; RDT, rapid diagnostic test.

**Table 2:** Qualitative data of MDA concentrations in malaria and uninfected controls.

No.	Author, Year	Study Location	Age Range (Years)	Study Findings
1	Akanbi et al., 2010 [52]	Nigeria	Not specified	MDA levels showed a significant positive correlation with parasite density (r = 0.53, *p* < 0.05).
2	Awalu et al., 2022 [34]	Nigeria	17–21 years	There were significantly increased levels of MDA in malaria, typhoid, malaria/typhoid, and peptic ulcer groups compared with healthy participants (*p* < 0.05).
3	Babalola et al., 2022 [35]	Nigeria	15–40 years	Differences in MDA levels between malaria-positive pregnant women and controls were not statistically significant (*p* > 0.05). The mean MDA level was significantly higher (*p* < 0.05) in malaria-positive primigravidae and secundigravidae than in multigravidae. The difference across the groups (control, mild, moderate, and severe) was not statistically significant (*p* > 0.05).
4	Bilgin et al., 2012 [36]	Turkey	15–46 years	MDA levels were significantly higher in patients with *P. vivax* malaria than in healthy controls (*p* < 0.05).
5	D’Souza et al., 2009 [37]	India	18–60 years	MDA levels were highly and significantly increased in both *P. vivax* and *P. falciparum* malaria patients (*p* < 0.001) compared with controls. The increase in MDA levels in *P. falciparum* malaria patients was much more than in *P. vivax* malaria patients.
6	Das et al., 1990 [38]	India	1–12 years	Plasma MDA levels were significantly higher in malaria patients than in control subjects (*p* < 0.001).
7	Erel et al., 1997 [53]	Turkey	15–35 years	MDA levels were significantly higher in malaria patients than in controls (*p* < 0.05).
8	Fabbri et al., 2013 [39]	Brazil	Not specified	Plasma MDA levels were significantly increased in malaria patients with and without jaundice compared with controls.
9	Farombi et al., 2003 [40]	Nigeria	18–35 years	MDA levels were significantly increased in malaria patients, uninfected patients treated with chloroquine, and malaria patients treated with chloroquine compared with controls.
10	Krishna et al., 2009 [41]	India	15–55 years	MDA levels were significantly increased in malaria patients compared with healthy controls (*p* < 0.05). MDA levels were significantly higher in patients with *P. vivax* malaria than in those with *P. falciparum* malaria (*p* < 0.05).
11	Megnekou et al., 2015 [7]	Cameroon	16–39 years	MDA levels were significantly higher in women with malaria than in uninfected women (*p* = 0.0047). The MDA levels also correlated positively with parasitemia (*p* = 0.0024).
12	Nanda et al., 2004 [42]	India	Not specified	MDA levels were significantly elevated in cases where *P. falciparum* malaria induced acute renal failure compared with uncomplicated *P. falciparum* malaria (*p* < 0.001) and healthy controls (*p* < 0.001). Serum MDA levels were positively correlated with urea (r = 0.62, *p* < 0.025), creatinine (r = 0.65, *p* < 0.05), and bilirubin (r = 0.72, *p* < 0.001) levels.
13	Narsaria et al., 2012 [43]	India	0–15 years	Plasma MDA levels were significantly raised in malaria cases compared with controls (*p* < 0.001).
14	Narsaria et al., 2015 [44]	India	0–16 years	Mean plasma MDA levels were significantly higher in patients with severe malaria compared with controls (*p* < 0.05).
15	Nsonwu-Anyanwu et al., 2019 [45]	Nigeria	18–60 years	MDA levels were higher in malaria patients with or without antimalarial therapy compared with controls (*p* < 0.05). Parasitemia and MDA levels were positively correlated (r = 0.399, *p* = 0.029) in malaria patients without antimalarial therapy.
16	Nwagha et al., 2011 [46]	Nigeria	21–30 years	Differences in serum MDA levels between malaria-negative and malaria-positive subjects were not statistically significant (1st trimester: *p* = 0.69, 2nd trimester: *p* = 0.68, 3rd trimester: *p* = 0.57; and control: *p* = 0.59).
17	Polat et al., 2002 [47]	Turkey	Not specified	MDA levels in malaria patients were higher than in controls (*p* < 0.001).
18	Raza et al., 2013 [54]	India	0.5–5 years	MDA levels in malaria patients were significantly higher compared with controls (*p* < 0.05).
19	Sakyi et al., 2012 [48]	Ghana	Children 10 years of age and below	MDA levels were higher in severe malaria patients compared with the controls and patients with uncomplicated malaria. MDA levels and malaria parasite density were positively correlated (r = 0.936, *p* < 0.05).
20	Tiyong Ifoue et al., 2009 [49]	Cameroon	16–44 years	MDA levels were significantly higher in patients compared with controls (*p* < 0.001). MDA levels were higher in primigravidae and correlated well with parasite density (*p* < 0.001).
21	Upadhyay et al., 2011 [50]	India	20–52 years	Serum MDA levels were significantly increased in malaria patients (*p* < 0.001). MDA levels were significantly increased in patients with *P. falciparum* compared with *P. vivax* malaria (*p* < 0.001).
22	Villaverde et al., 2020 [51]	Ugandan	1.5–11.7 years	Differences in MDA levels between malaria cases and controls were not statistically significant.
23	Wankasi et al., 2020 [55]	Nigeria	18–65 years	MDA levels were significantly increased (*p* < 0.05) in malaria patients compared with the control group.

Abbreviations: MDA, malondialdehyde.

## Data Availability

All data relating to the manuscript were found in the main texts and Supplementary files.

## Supplementary Materials

The following supporting information can be downloaded at: https://www.mdpi.com/article/10.3390/antiox12081502/s1, Table S1. Search terms; Table S2. Details of the included studies; Table S3. Quality of the included studies; Table S4. Meta-regression results; PRISMA Abstract Checklist; PRISMA 2020 Checklist.

## Abbreviations

CI, confidence interval; MDA, malondialdehyde; PCR, polymerase chain reaction; PICO, patient, intervention, comparison, outcome; RDT, rapid diagnostic test; ROS, reactive oxygen species; SOD, superoxide dismutase; STROBE, Strengthening the Reporting of Observational Studies in Epidemiology.
