# Supplementary material for: Increased Blood Concentrations of Malondialdehyde in Plasmodium Infection: A Systematic Review and Meta-Analysis
Source: Antioxidants (Basel). 2023 Jul 27;12(8):1502. doi: 10.3390/antiox12081502 (PMC10452025; doi:10.3390/antiox12081502)
Supplement: Supplementary file 1 [file antioxidants-12-01502-s001.zip › Table S1. Search terms.docx]

**Table S1. Search strategy**

**PubMed**

**18 January 2023**

| **No.** | **Query** | **Results** |
| --- | --- | --- |
| 3 | #1 AND #2 | 125 |
| 2 | (((malaria) OR (malaria[MeSH Terms])) OR (Plasmodium)) OR (Plasmodium[MeSH Terms]) | 120,504 |
| 1 | (Malonyldialdehyde) OR (Malonyldialdehyde[MeSH Terms]) | 68,815 |

**Scopus**

**18 January 2023**

| **No.** | **Query** | **Results** |
| --- | --- | --- |
| 3 | 1 AND 2 | 167 |
| 2 | TITLE-ABS-KEY ( malaria OR plasmodium OR "remittent fever" OR "marsh fever" OR paludism ) | 154,039 |
| 1 | TITLE-ABS-KEY ( propanedial OR malonyldialdehyde OR malonaldehyde OR malonylaldehyde OR "sodium malondialdehyde" ) | 73,417 |

**MEDLINE**

| **No.** | **Search terms/Search strategy** | **Date** |
| --- | --- | --- |
| 1 | (Propanedial OR Malonyldialdehyde OR Malonaldehyde OR Malonylaldehyde OR “Sodium Malondialdehyde”) AND (malaria OR Plasmodium OR "Remittent Fever" OR "Marsh Fever" OR Paludism)  Search results: 34 | **18 January 2023** |

**Embase**

**18 January 2023**

| **No.** | **Query** | **Results** |
| --- | --- | --- |
| 3 | #1 AND #2 | 191 |
| 2 | malaria OR plasmodium OR 'remittent fever' OR 'marsh fever' OR paludism | 160,525 |
| 1 | propanedial OR 'malonyldialdehyde'/exp OR malonyldialdehyde OR 'malonaldehyde'/exp OR malonaldehyde OR 'malonylaldehyde'/exp OR malonylaldehyde OR 'sodium malondialdehyde' | 80,944 |

**ProQuest**

| **No.** | **Search terms/Search strategy** | **Date** |
| --- | --- | --- |
| 1 | (Propanedial OR Malonyldialdehyde OR Malonaldehyde OR Malonylaldehyde OR “Sodium Malondialdehyde”) AND (malaria OR Plasmodium OR "Remittent Fever" OR "Marsh Fever" OR Paludism)  Search results: 42 | **18 January 2023** |

**Ovid**

| **No.** | **Search terms/Search strategy** | **Date** |
| --- | --- | --- |
| 1 | (Propanedial OR Malonyldialdehyde OR Malonaldehyde OR Malonylaldehyde OR “Sodium Malondialdehyde”) AND (malaria OR Plasmodium OR "Remittent Fever" OR "Marsh Fever" OR Paludism) {No Related Terms}  Search results: 282 | **18 January 2023** |
