# Supplementary material for: Increased Blood Concentrations of Malondialdehyde in Plasmodium Infection: A Systematic Review and Meta-Analysis
Source: Antioxidants (Basel). 2023 Jul 27;12(8):1502. doi: 10.3390/antiox12081502 (PMC10452025; doi:10.3390/antiox12081502)
Supplement: Supplementary file 1 [file antioxidants-12-01502-s001.zip › Table S4. Meta-regression results.docx]

**Table S4. Meta-regression results**

| **Meta-analysis of MDA** | **Covariates** | **P value** | **R-squared (%)** | **tau2** | **I^2^ (%)** | **Number of study** |
| --- | --- | --- | --- | --- | --- | --- |
| **Malaria vs uninfected controls** | Publication years | 0.09 | 3.06 | 1.26 | 97.8 | 14 |
|  | Study design | N/A | N/A | N/A | N/A | N/A |
|  | Continent | 0.64 | 18.9 | 1.05 | 94.9 | 14 |
|  | Participants ‘group | 0.33 | 0 | 1.43 | 95.9 | 14 |
|  | Age group | 0.93 | 0.75 | 1.14 | 96.01 | 12 |
|  | *Plasmodium* spp. | 0.06 | 12.5 | 0.59 | 89.8 | 10 |
|  | Method for malaria detection | 0.14 | 0 | 0.75 | 91.2 | 10 |
|  | Quality of study | 0.05 | 12.6 | 2.13 | 95.8 | 14 |

N/A, not assessed because of collinearity
